# Supplementary material for: Combined Phyllostachys pubescens and Scutellaria baicalensis Prevent High-Fat Diet-Induced Obesity via Upregulating Thermogenesis and Energy Expenditure by UCP1 in Male C57BL/6J Mice
Source: Nutrients. 2022 Jan 19;14(3):446. doi: 10.3390/nu14030446 (PMC8840647; doi:10.3390/nu14030446)
Supplement: Supplementary file 1 [file nutrients-14-00446-s001.zip › nutrients-1542138-supplementary.pdf]

nutrients

MDPI

Table S1. The used primer and probe sequences.

Gene	Forward	Reverse
Cebpa	TGGACAAGAACAGCAACGAGTAC	CGGTCATTGTCACTGGTCAACT
Fasn	CTGAGATCCCAGCACTTCTTGA	GCCTCCGAAGCCAAATGAG
Srebf1	AGCCTGGCCATCTGTGAGAA	CAGACTGGTACGGGCCACAA
Pparg	TCGGAATCAGCTCTGTGGACCTCTCC (FAM)	
Lep	CCAAAACCCTCATCAAGACC	GTCCAACTGTTGAAGAATGTCCC
Ppara	CGACCTGAAAGATTCGGAAA	CTTTCCCGCGAGTATGACC
Ucp2	CCGCATTGGCCTCTACGACTCT	CCCCGAAGGCAGAAGTGAAGTG
Adipoq	CCCAAGGGAACTTGTGCAGGTTGGATG	GTTGGTATCATGGTAGAGAAGAAAGCC
Ucp1	CGACTCAGTCCAAGAGTACTTCTCTTC	GCCGGCTGAGATCTTGTTTC
Cpt1b	GTCGCTTCTTCAAGGTCTGG	AAGAAAGCAGCACGTTCGAT
Sirt1	GTTCTGACTGGAGCTGGGGT	ATGGCTTGAGGATCTGGGAG
Ppargc1a	GCACCAGAAAACAGCTCCAA	TTACTGAAGTTGCCATCCCG
Fndc5	ATGAAGGAGATGGGGAGGAA	GCGGCAGAAGAGAGCTATAACA
Gapdh	TGCATCCTGCACCACCAACTGCTTAG (VIC)	
